# Progranulin Is a Novel Independent Predictor of Disease Progression and Overall Survival in Chronic Lymphocytic Leukemia

**DOI:** 10.1371/journal.pone.0072107

**Published:** 2013-08-23

**Authors:** Maria Göbel, Lewin Eisele, Michael Möllmann, Andreas Hüttmann, Patricia Johansson, René Scholtysik, Manuela Bergmann, Raymonde Busch, Hartmut Döhner, Michael Hallek, Till Seiler, Stephan Stilgenbauer, Ludger Klein-Hitpass, Ulrich Dührsen, Jan Dürig

**Affiliations:** 1 Department of Hematology, University Hospital, University of Duisburg-Essen, Essen, Germany; 2 Institute for Medical Informatics, Biometry and Epidemiology, University of Duisburg-Essen, Essen, Germany; 3 Institute of Cell Biology, University Hospital, University of Duisburg-Essen, Essen, Germany; 4 Department of Internal Medicine III, University of Ulm, Ulm, Germany; 5 Institute for Medical Statistics and Epidemiology, Technical University Munich, Munich, Germany; 6 Department I of Internal Medicine, University Hospital Cologne, and Center of Integrated Oncology Köln-Bonn, Köln, Germany; 7 Department of Medicine III, University Hospital Großhadern, Munich, Germany; Westmead Millennium Institute, University of Sydney, Australia

## Abstract

Progranulin (Pgrn) is a 88 kDa secreted protein with pleiotropic functions including regulation of cell cycle progression, cell motility, wound repair and tumorigenesis. Using microarray based gene expression profiling we have recently demonstrated that the gene for Pgrn, granulin (*GRN)*, is significantly higher expressed in aggressive CD38^+^ZAP-70^+^ as compared to indolent CD38^−^ZAP-70^−^ chronic lymphocytic leukemia (CLL) cases. Here, we measured Pgrn plasma concentrations by enzyme-linked immunosorbent assay (ELISA) in the Essen CLL cohort of 131 patients and examined Pgrn for association with established prognostic markers and clinical outcome. We found that high Pgrn plasma levels were strongly associated with adverse risk factors including unmutated IGHV status, expression of CD38 and ZAP-70, poor risk cytogenetics (11q-, 17p-) as detected by flourescence in situ hybridization (FISH) and high Binet stage. Pgrn as well as the aforementioned risk factors were prognostic for time to first treatment and overall survival in this series. Importantly, these results could be confirmed in the independent multicentric CLL1 cohort of untreated Binet stage A patients (n = 163). Here, multivariate analysis of time to first treatment revealed that high risk Pgrn (HR = 2.06, 95%-CI = 1.13–3.76, p = 0.018), unmutated IGHV status (HR = 5.63, 95%-CI = 3.05–10.38, p<0.001), high risk as defined by the study protocol (HR = 2.06, 95%-CI = 1.09–3.89, p = 0.026) but not poor risk cytogenetics were independent prognostic markers. In summary our results suggest that Pgrn is a novel, robust and independent prognostic marker in CLL that can be easily measured by ELISA.

## Introduction

Chronic lymphocytic leukemia (CLL) is a heterogenous disease with a highly variable clinical course. In a continuing effort to investigate a molecular basis that may underlie this diverse clinical behavior and discover novel prognostic molecular markers we have performed a series of gene expression profiling studies using DNA microarray technology [1]–[3]. Comparing the transcriptomes of ZAP-70^+^CD38^+^ *vs.* ZAP-70^−^CD38^−^ patients representing the extremes of the disease spectrum, we identified a panel of CLL subtype distinction genes, the majority of which was found to be over-expressed in the prognostically unfavorable ZAP-70^+^CD38^+^ subgroup [2], [3]. Following up on these results we then further investigated a number of candidate genes for their prognostic value and potential functional relevance to the disease process [4]–[6].

Our more recent work focused on progranulin (Pgrn) which is a secreted glycoprotein identified as one of the top 20 CLL subtype distinction genes over-expressed in ZAP-70^+^CD38^+^ cases [2]. Pgrn is encoded by the gene granulin (*GRN)* located on chromosome 17q21.31 and consists of tandem repeats of a 12-cysteine module termed granulin [7], [8]. *GRN* is expressed in a wide range of different tissues including bone marrow stromal cells and elements of the innate and adaptive immune system [9]. The progranulin protein exhibits pleiotropic functions as a regulator of cell proliferation, survival and migration under both physiological and pathologic conditions [7], [8]. Pgrn has been described as a neurotrophic factor and inactivating *GRN* mutations cause frontotemporal lobar degeneration, which is a devastating neurological disease characterized by the presence of ubiquitinated inclusions of the transactivation response element DNA-binding protein-43 [10]. By contrast, elevated Pgrn levels have been associated with a wide range of different human malignancies. There is now accumulating evidence that Pgrn acts as a tumor promoting factor in carcinomas of the breast [11], ovary [12] liver [13], kidney [14], prostate [15] and the brain [16]. Furthermore, high Pgrn expression levels as detected in the tumor itself or in the peripheral blood have been linked to an aggressive phenotype and poor prognosis in breast cancer [17], glioblastoma [16] and ovarian cancer [18].

However, as yet data on the role of Pgrn in hematological malignancies are limited to multiple myeloma where it has been demonstrated *in vitro* to promote cell survival and confer resistance to dexamethasone treatment [19], [20]. The objective of this study was to confirm and expand our microarray results regarding differential *GRN* expression in high *vs.* low risk CLL subgroups and explore its potential as a novel prognostic factor. To this end, we measured Pgrn concentrations in plasma and serum samples from CLL patients and investigated the relationship of Pgrn levels with established prognostic markers and clinical outcome.

## Patients, Materials and Methods

### Patients

Unicentric cohort from Essen: Peripheral blood samples from 131 patients with CLL and 31 healthy blood donors were analyzed after obtaining written informed consent according to our institutional guidelines. This study was approved by the Ethics Commission of the University of Duisburg-Essen (reference 04–2459). Plasma samples were freshly prepared by centrifugation at 600 G for 5 min within four hours after blood collection and stored at −80°C until analysis. The diagnosis of CLL required a persistent lymphocytosis of more than 5.0×10^9^/l and a typical CD19^+^, CD20^+^ CD5^+^, CD23^+^, Ig light chain (κ or λ light chain) restricted immunophenotype as revealed by flow cytometry of peripheral blood cells [21]. Patient selection was based on the availability of plasma samples from our local CLL biobank. At the time of sample collection 28.2% of the patients had previously received chemotherapy but had been left untreated for a minimum of 12 months and 71.8% of the patients were treatment-naïve. For the patients that had received prior therapy the date of sample collection was defined as the starting point for the follow-up period. The median time between first diagnosis and sample collection was 22 months (range 0 to 302 months). The following patient characteristics were collected and analyzed: clinical stage according to Binet, IGHV mutational status (unmutated:≥98% homology; mutated<98% homology to the germ line sequence), cytogenetics determined by flourescence in situ hybridization (FISH), CD38 expression (negative<30%; positive≥30%), ZAP-70 expression as detected by flow cytometry (negative:<20%; positive≥20%) and percentage of smudge cells on blood smears [22] along with routine laboratory data. Molecular risk factors were determined as previously described [5].

Validation cohort provided by the German CLL study group (GCLLSG): The German CLL study group kindly provided serum samples of 163 previously untreated Binet stage A patients, representing a subgroup of the risk-stratified CLL1 trial of the GCLLSG (clinicaltrials.gov: NCT00262782). Median follow-up of this subgroup was 6.6 years. Serum samples had been collected centrally at study entry and stored at −80°C. All patients provided written informed consent for use of their serum samples for clinical and research purposes. This study was approved by Ethics Commission of the University of Duisburg-Essen (reference number 99-184-1315-Y). The clinical characteristics and laboratory data of the two study populations are shown in Tables 1 and 2.

**Table 1:** Patient characteristics of the CLL cohort from Essen.

		Total	Progranulin low (≤165.5 ng/ml)	Progranulin high (>165.5 ng/ml)	p
**Age**	N	131	66	65	0.542
	mean ± SD	62.02±11.11	61.42±10.88	62.62±11.40	
	median (range)	63(31–87)	63 (37–83)	63 (31–87)	
**Sex**	Male	86 (65.6%)	40 (46.5%)	46 (53.5%)	0.217
	Female	45 (34.4%)	26 (57.8%)	19 (42.2%)	
**Binet at diagnosis**	A	91 (69.5%)	53 (58.2%)	38 (41.8%)	**0.027**
	B	24 (18.3%)	7 (29.2%)	17 (70.8%)	
	C	15 (11.5%)	6 (40%)	9 (60%)	
	Missing	1 (0.8%)			
**IGHV**	unmutated	33 (25.2%)	8 (24.2%)	25 (75.8%)	**<0.001**
	mutated	37 (28.2%)	26 (70.3%)	11 (29.7%)	
	missing	61 (46.6%)			
**CD38**	negative (<30%)	68 (51.9%	40 (58.8%)	28 (41.2%)	0.107
	positive (>30%)	58 (44.3%)	25 (43.1%)	33 (56.9%)	
	missing	5 (3.8%)			
**ZAP-70**	negative (<20%	31(23.7%)	21 (67.7%)	10 (32.3%)	**0.027**
	positive (>20%)	36 (27.5%)	14 (38.9%)	22 (61.1%)	
	missing	64 (48.9%)			
**FISH risk group** *	low risk	96 (73.3%)	60 (62.5%)	36 (37.5%)	**<0.001**
	high risk	33 (25.2%)	6 (18.2%)	27 (81.8%)	
	missing	2 (1.5%)			
**β2-microglobulin**	N	74 (56.5%)	37	37	**<0.001**
	mean ± SD	3.46±2.18	2.45±0.90	4.47±2.59	
	median (range)	2.6 (1.3–11.7)	2.35 (1.30 - 4.70)	3.40 (1.93–11.70)	
**Leucocytes**	N	85 (64.9%)	43	42	0.188
	mean ± SD	57.67±76.76	46.8±84.51	68.79±67.13	
	median (range)	26.8 (0.3–539.0)	20.40 (5.9–539.0)	42.9 (0.3–272.0)	
**Therapy**	therapy	73 (55.7%)	24 (32.9%)	49 (67.1%)	**<0.001**
	no therapy	58 (44.3%)	42 (72.4%)	16 (27.6%)	
**Survival**	Dead	27 (20.6%)	5 (18.5%)	22 (81.5%)	**<0.001**
	Alive	104 (79.4%)	61 (58.7%)	43 (41.3%)	

* Leukemic samples exhibiting a 17p- and/or 11q- karyotype were assigned to the high risk and samples with either no chromosomal abnormalities or aberrations of chromosomes 13q and 12 to the low risk category. Leukocyte counts and age were assessed at the time of sample acquisition whereas all other risk parameters were determined at the time of diagnosis.

**Table 2:** Patient characteristics of the CLL1 study cohort.

		Total	Progranulin low (≤79.2 ng/ml)	Progranulin high (>79.2 ng/ml)	p
**Age**	N	163	82	81	0.967
	mean ± SD	59.94±8.22	60.21±7.95	59.68±8.53	
	median (range)	61 (35–75)	60.5 (35–75)	62 (37–75)	
**Sex**	Male	96 (58.9%)	49 (51.0%)	47 (49.0%)	0.628
	female	62 (38.0%)	29 (46.8%)	33 (53.2%)	
	missing	5 (3.1%)			
**Study arm** *	low risk	138 (84.7%)	76 (55.1%)	62 (44.9%)	**0.008**
	high risk	24 (14.7%)	6 (25%)	18 (75%)	
	missing	1(0.6%)			
**IGHV**	Unmutated	42 (25.8%)	11 (26.2%)	31 (73.8%)	**<0.001**
	mutated	121 (74.2%)	71 (58.7%)	50 (41.3%)	
**FISH risk group** **	low risk	153 (93.9%)	81 (52.9%)	72 (47.1%)	**0.009**
	high risk	10 (6.1%)	1 (10%)	9 (90%)	
**β2-microglobulin**	N	163	82 (49.3%)	81 (50.7%)	**0.001**
	mean ± SD	1.67±0.59	1.52±0.40	1.81±0.70	
	median (range)	1.53 (0.90–6.2)	1.5 (0.9–3.1)	1.68 (0.95–6.2)	
**Thymidine kinase**	N	163	82	81	**0.002**
	mean ± SD	7.49±6.05	5.97±3.68	9.02±7.47	
	median (range)	5.5 (2.0–34.1)	5.15 (2.0–20.4)	6.5 (2.0–34.1)	
**Leucocytes**	N	133 (82.6%)	68 (51.1%)	65 (48.9%)	**<0.001**
	mean ± SD	27.6±23.7	20.8±9.4	34.8±31.1	
	median (range)	21.6 (4.7–184.0)	18.4 (6.3–66.0)	26.4 (4.7–184.0)	
**Lymphocyte doubling time (LDT)**	<12 months	27 (16.6%)	11 (40.7%)	16 (59.3%)	0.299
	≥12 months	134 (82.2%)	70 (52.2%)	64 (47.8%)	
	missing	2 (1.2%)			
**LDH blood level**	N	146 (89.6%)	72 (49.3%)	74 (50.7%)	**0.006**
	mean ± SD	195.1±71.8	178.5±46.3	211.3±87.3	
	median (range)	178 (82–732)	169.5 (82–332)	183 (94–732)	
**Therapy**	therapy	55 (33.7%)	17 (30.9%)	38 (69.1%)	**<0.001**
	no therapy	108 (66.3%)	65 (60.2%)	43 (39.8%)	
**Survival**	Dead	20 (12.3%)	3 (15%)	17(85%)	**0.001**
	alive	143 (87.7%)	79 (55.2%)	64 (44.8%)	

* high risk, as defined by the following: serum thymidine kinase level>7.0 U/L, β2-microglobulin>3.5 mg/l level, presence of non-nodular bone marrow infiltration, LDT<12 months; low risk: none of the criteria listed for high risk disease.

** For definition of cytogenetic risk see legend to Table 1. All risk parameters except LDT were determined at the time of study entry.

### Quantification of Progranulin Levels in Plasma and Serum Samples by Enzyme-linked Immunosorbant Assay (ELISA)

Progranulin plasma (Essen cohort), serum (CLL1 validation cohort) and cell culture supernatant levels were measured using Quantikine Kits, according to the manufacturer`s instructions (R&D Systems, Wiesbaden, Germany). The absorbance was recorded by a MRX microplate reader and analyzed by Revelation software version 4.22 (Dynatech Laboratories, Denkendorf, Germany). Intra- and inter-plate assay precision determined by coefficients of variance (CV) were 4.9% and 7.9%, as reported by the manufacturer.

### Cell Cultures

Leukemic B-cells were isolated from the PB of CLL patients employing Lymphoprep (Invitrogen, Karlsruhe, Germany) density-gradient centrifugation resulting in a purity of >90% as determined by flow cytometry. CLL cells were cultured at a concentration of 1.5×10^6^/ml in RPMI1640 (Sigma, Taufkirchen, Germany) with 10% (v/v) fetal calf serum (FCS, PAA, Cölbe, Germany) supplemented with 1000 U/ml penicillin and 100 U/ml streptomycin (both from Sigma, Taufkirchen, Germany) in the absence (controls) or presence of different concentrations of human recombinant progranulin (10, 100 and 1000 ng/ml; R&D Systems, Wiesbaden, Germany). After two and five days incubation in a 5% CO_2_ and air incubator at 37°C cell culture supernatants from the control samples were frozen at −80°C until Pgrn measurement as described above. Cell viability was assessed using trypan blue exclusion.

### Statistical Analysis

Comparisons of clinical and laboratory parameters between patient subgroups were performed using the Mann-Whitney U test (for continuous quantitative variables) and the Fisher’s exact test (for categorical variables). Spearman correlation coefficients were calculated to analyze the correlation between two continuous variables. Survival time and censored waiting times measured from time of diagnosis were plotted by the Kaplan-Meier method and compared using the log-rank test. For multivariate analysis we used a Cox proportional hazards model. The statistical analyses were performed with SPSS statistics version 20 (IBM, Ehningen, Germany) and GraphPad Prism version 6 (GraphPad Software, Inc., La Jolla, USA).

## Results

### Progranulin (Pgrn) Plasma Concentrations in Normal Individuals and CLL Patients

**Figure 1:**
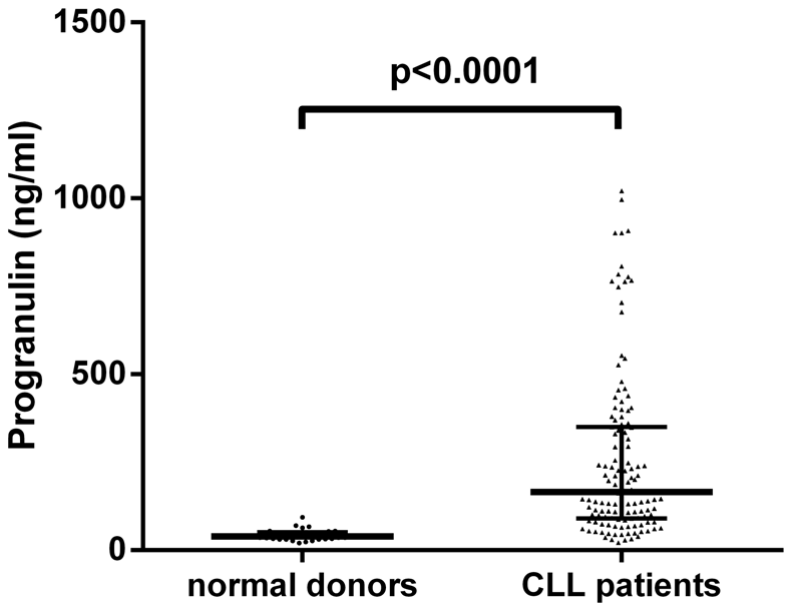
Progranulin plasma levels in CLL patients and normal blood donors. ELISA analysis of plasma samples collected from CLL patients (N = 131) and normal blood donors (ND, N = 31) reveals statistically significant differences in Pgrn concentrations (Mann-Whitney U-Test). The bold horizontal bars represent the median and the whiskers indicate the interquartile range.

We determined the concentration of Pgrn in plasma samples from 31 normal individuals and 131 CLL patients by ELISA. CLL patients exhibited significantly elevated Pgrn levels (median: 165.5 ng/ml, range: 20.8–1021.6 ng/ml) as compared to normal controls (median: 38.4 ng/ml, range: 20.0–93.0 ng/ml; Figure 1) with no apparent differences for age and sex (p>0.05, data not shown). To investigate the relationship between *GRN* mRNA levels in CLL cells and protein secretion we quantified the concentration of Pgrn in plasma samples from 16 CLL patients with available gene expression data from a previous microarray study [2]. As shown in Figure S1, a statistically significant positive correlation of the two parameters (p = 0.0077, R^2^ = 0.4329) could be observed. Furthermore, cell culture experiments revealed that the leukemic cells are capable of secreting Pgrn into the culture supernatant in a time-dependent fashion with large interindividual differences (Figure S2A). These findings together with the microarray data provide evidence that the CLL cells are indeed the source of excess Pgrn protein production in CLL patients. Aiming at a potential tumor-promoting effect of Pgrn at the cellular level which was recently reported in cholangiocarcinoma [23] and prostate cancer cells [15] we assessed the viability and number of CLL cells cultured for two to five days in the absence or presence of different concentrations (10–1000 ng/ml) of recombinant Pgrn. As depicted in Figure S2B we could not detect a pro-survival effect of Pgrn on CLL cells under these experimental conditions.

### Correlation of Progranulin and Established Prognostic Markers in CLL

**Figure 2:**
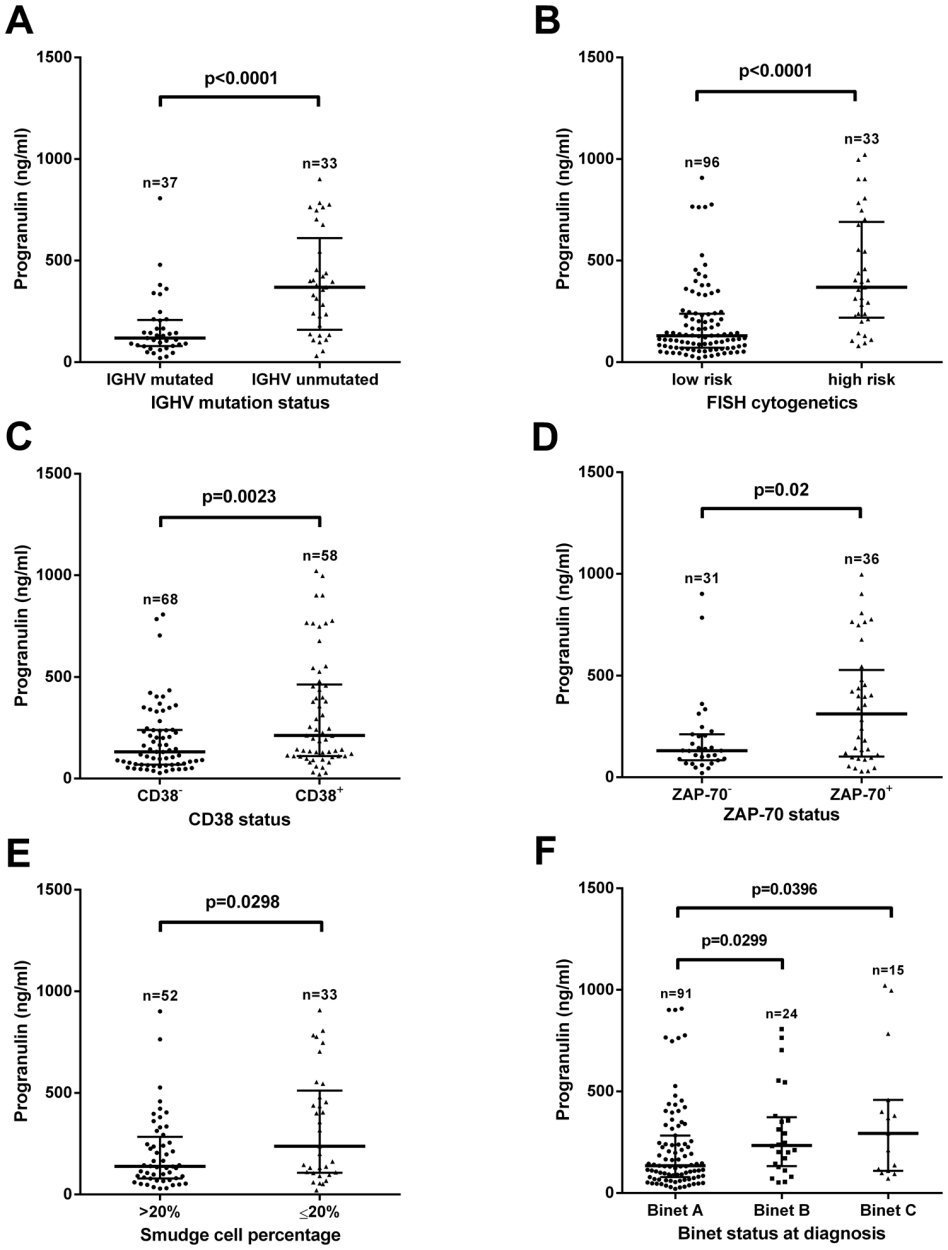
Correlation of Progranulin plasma levels and established prognostic markers in the CLL cohort from Essen. Plasma samples collected from high-risk patients defined by the presence of unmutated IGHV genes (A), presence of del17p and/or del11q as detected by iFISH (B), expression of CD38 (C) and ZAP-70 (D), low percentage of smudge cells (E) as well as advanced Binet stage B/C (F) exhibited significantly higher Pgrn plasma concentrations as compared to low-risk patients. Statistical comparisons were made using the Mann-Whitney U-test (A–E) and Kruskall Wallis test (F). The bold horizontal bars represent the median and the whiskers indicate the interquartile range.

The distribution of Progranulin plasma levels in the study populations is shown in Figure S3. For analysis of correlation between Pgrn and clinical prognostic markers, CLL patients from Essen were separated into two groups using the median Pgrn plasma concentration of 165.5 ng/ml as a cut-off (Figure S3A). We observed a strong association between high Pgrn plasma levels and poor prognostic markers, such as Binet disease stage at diagnosis, IGHV mutational status, ZAP-70, CD38, low percentage of smudge cells and high risk cytogenetic aberrations (11q-, 17p-, Figure 2).

### Association between Pgrn and Clinical Outcome

**Figure 3:**
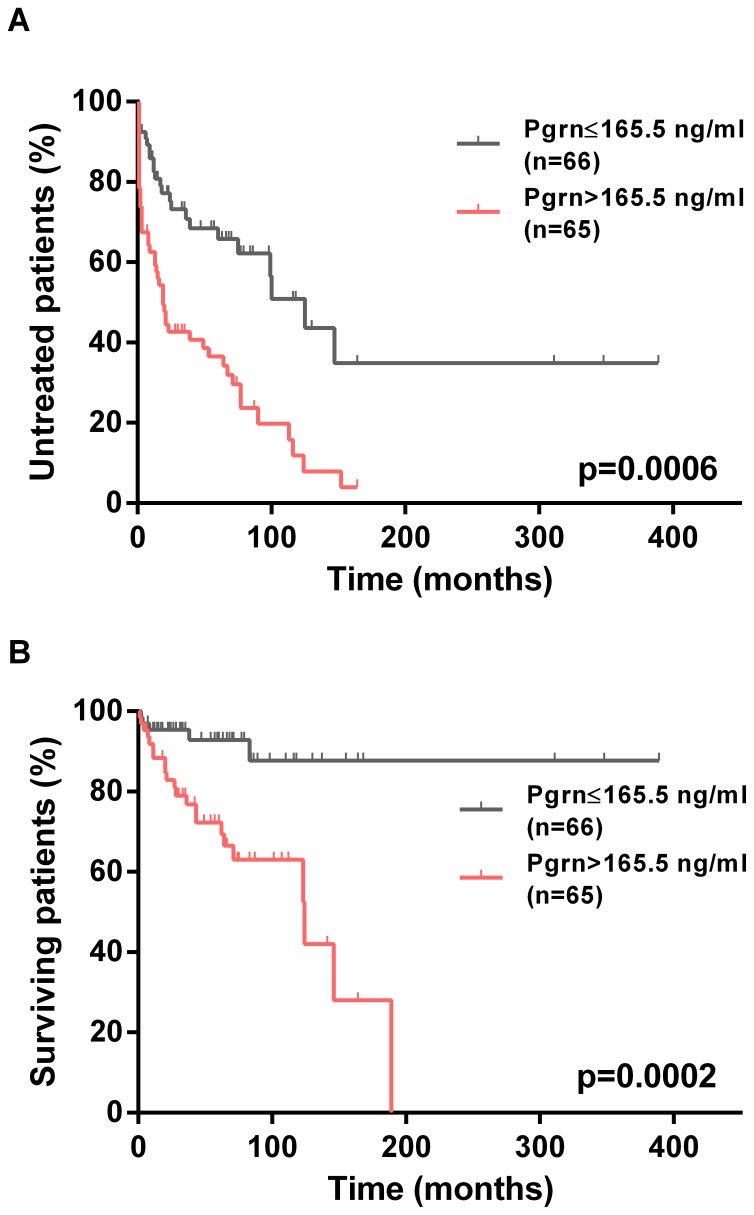
Association of Pgrn plasma levels and clinical outcome in the CLL cohort from Essen. Kaplan-Meier curves depict the cumulative proportion of untreated (TTFT, A) and surviving (OS, B) patients with CLL. Statistical comparisons between patients with high (>165.5 ng/ml) and low Pgrn plasma levels (≤165.5 ng/ml) were performed using the log-rank test.

Of the 131 patients from the Essen cohort, the median follow-up was 54 months (range 1–389 months) and 73 patients required treatment according to National Cancer Institute Working Group criteria. The median time from diagnosis to first treatment (TTFT) of the entire cohort was 64 months (95% CI, 32–96 months). As shown in Figure 3A, the median TTFT in patients with high Pgrn plasma levels (19 months) was significantly shorter than in patients with low Pgrn concentrations (125 months, p<0.0001). In line with these data on disease progression (Figure 3A), we also observed statistically highly significant differences in terms of overall survival (OS) between the two groups. The median survival of Pgrn high patients was 124 months, whereas the median survival for the Pgrn low subgroup was not reached during follow-up (p = 0.0003, Figure 3B). For comparison, Figure S4 shows Kaplan-Meier curves for established prognostic markers analyzed in this cohort.

### Time Course Analysis of Pgrn Plasma Levels

**Figure 4:**
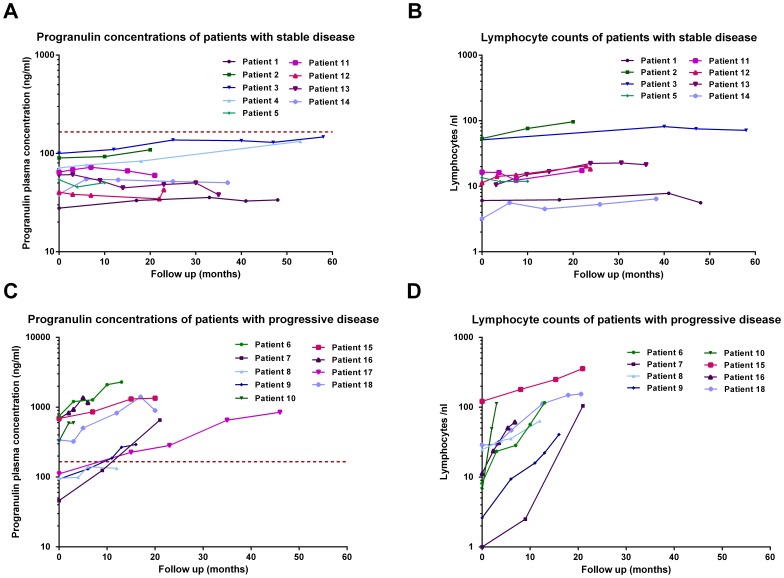
Time course analysis of Pgrn plasma levels and PB lymphocyte counts in stable *vs.* progressive CLL patients. Sequential PB lymphocyte counts and corresponding Pgrn plasma levels in serial samples of nine individuals with stable (A and B) and nine patients with progressive disease (C and D). Lines connect the symbols of individual patients. The horizontal red dotted line in A and C represents the median Pgrn plasma concentration of the Essen CLL cohort.

Eighteen patients from the Essen cohort, nine individuals with stable and nine patients with progressive disease (defined by increasing lymphocyte counts and requiring therapy) were studied at three or more time-points (range 3–5). As illustrated in Figure 4, serial Pgrn plasma levels varied substantially between patient subgroups, showing a clear positive association between increasing leukemic cells and Pgrn plasma levels.

### Validation Experiments in an Independent Cohort of Binet Stage A Patients

In a second set of experiments we aimed to confirm our findings in an independent multicentric prospectively followed patient series. To this end we employed serum samples collected from a representative subset of Binet stage A patients included in the CLL1 study of the German CLL study group (GCLLSG). In a pilot series of N = 5 patients from the Essen cohort we could not detect relevant differences in Pgrn levels measured in heparin plasma as compared to serum collected simultaneously from the same patient (p>0.05, Figure S5).

Comparing Pgrn blood levels in the CLL1 cohort with our unicentric results from Essen revealed a largely similar distribution pattern, however the median Pgrn concentration was significantly higher in the latter patient group (Figure S3, p<0.0001).

**Figure 5:**
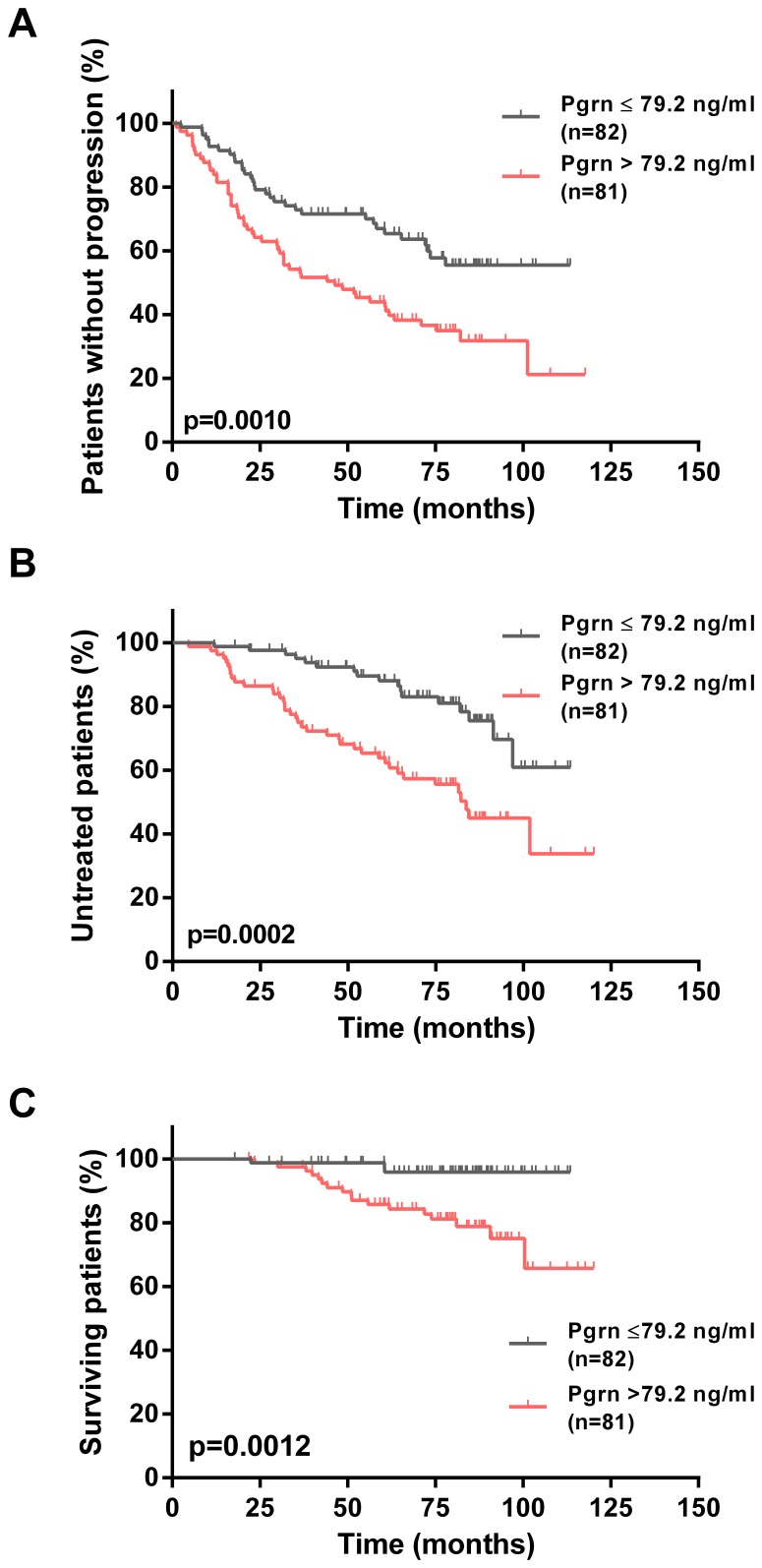
Association of Pgrn plasma levels and clinical outcome in the multicentric CLL1 cohort. Kaplan-Meier curves depict the cumulative proportion of progression free survival (PFS, A), time to first treatment (TTFT, B) and overall survival (OS, C) of patients with CLL. Statistical comparisons between patients with high (>79.2 ng/ml) and low Pgrn plasma levels (≤79.2 ng/ml) were performed using the log-rank test.

Using the same approach as described for the Essen cohort, we then dichotomized the CLL1 patients into two groups with high or low Pgrn serum levels based on the median of 79.2 ng/ml. Univariate evaluation of established prognostic factors in these two groups revealed a strong association with adverse prognostic features thereby validating our findings in the unicentric cohort (Figure S6). Finally, we compared the clinical outcome of Pgrn high *vs.* low patients. Again, in line with our previous results, we found significantly shorter progression free survival (PFS), TTFT and OS times in CLL patients with high Pgrn serum levels as compared to their low Pgrn counterparts (Figure 5).

### Multivariate Analysis

Using multivariate Cox regression analysis for TTFT, we compared Pgrn with other prognostic factors. High Pgrn levels had independent power predicting an unfavorable prognosis in both patient cohorts (Table 3 and Table 4).

**Table 3:** Multivariate Cox regression analysis of TTFT for the CLL cohort from Essen.

N = 124	HR	95% CI	p
Progranulin (ng/ml)	1.003	1.001–1.004	<0.001
Binet at diagnosis A vs. B/C	2.493	1.473–4.219	0.001
CD38 expression	2.170	1.268–3.714	0.005
FISH risk group	1.688	0.912–3.125	0.096

**Table 4:** Multivariate Cox regression analysis of TTFT for the CLL1 study cohort.

N = 162	HR	95% CI	p
Progranulin (ng/ml)	1.003	1.001–1.004	0.003
Study arm (high risk)*	1.864	0.940–3.699	0.075
FISH risk group	1.418	0.582–3.455	0.442
IGHV mutation status	4.832	2.502–9.332	<0.001
LDH (U/l)	1.003	1.000–1.007	0.056

* For definition of high risk according to the CLL1 study protocol refer to legend to Table 2.

## Discussion

Following up on gene expression profiling studies from our own group [2] and recently published work of others [17], [24] we aimed to investigate the potential of progranulin as a novel prognostic biomarker in CLL. In line with data from studies in normal individuals [25], [26] and patients with frontotemporal lobar degeneration [27], breast [17] and ovarian cancer [18], we found that Pgrn can be easily and reliably measured in the peripheral blood employing a commercially available enzyme-linked immunosorbent assay. Pairwise comparisons of fresh *vs.* thawed (data not shown) and plasma *vs.* serum samples collected from individual patients yielded remarkably similar results, suggesting that the assay used in our study was very robust. These technical aspects compare favorably with some of the established prognostic markers in CLL which are more labor- and cost-intensive (IGHV mutation status, cytogenetics) [28] or difficult to standardize for routine assessment (ZAP-70) [29]. As a potential limitation common to most prognostic serum markers [30], it may be argued that Pgrn is not specific for CLL, but is secreted by a wide range of different tissues including bone marrow stromal cells. To address this issue at least in part, we compared *GRN* mRNA concentrations in immunomagnetically purified CLL cells with Pgrn protein plasma levels in the same patients and observed a highly significant correlation. Furthermore, cell culture studies using purified CLL cells revealed a time dependent secretion of Pgrn into the culture supernatant. These results provide circumstantial evidence that progranulin concentrations measured in the plasma indeed reflect the amount of progranulin production in the leukemic cells derived from individual patients.

Next, we evaluated the prognostic value of Pgrn in our local CLL cohort using the median Pgrn plasma level of 165.5 ng/ml to define two patient subgroups with low- *vs.* high risk disease. We found strong associations between high Pgrn plasma levels and poor prognostic markers, such as Binet disease stage at diagnosis, mutational status, ZAP70, CD38, low smudge cell counts and high risk cytogenetic aberrations (11q-, 17p-, Figure 1). Consistent with these findings, Kaplan-Meier analyses revealed significantly shorter TTFT and OS times in the Pgrn high *vs.* low patient subgroups. In order to validate these results we then measured Pgrn serum concentrations in an independent series of patients included in the CLL1 prospective multicenter trial of the German CLL study group (GCLLSG). Different from our own unicentric retrospective cohort, the CLL1 trial only included previously untreated Binet stage A patients. The median time from diagnosis to study entry was one year and the median follow-up of this series was 80 months. Comparing the distribution of progranulin levels in the two patient cohorts revealed a largely similar pattern, however the median Pgrn value was significantly lower in the CLL1 series as compared to the Essen cohort. This finding is probably due to the fact that about 30 percent of the Essen patients had advanced Binet stage B and C disease whereas only stage A patients were included into the CLL1 study. The more benign clinical characteristics of the CLL1 series is also exemplified by the longer time interval from diagnosis to first treatment as compared to the Essen patients (TTFT 101 months *vs.* 64 months) and the lower prevalence of adverse prognostic factors including FISH cytogenetics, IGHV and β2-microglobulin. Notwithstanding these differences, we observed the same associations between high Pgrn serum levels, poor prognostic markers, TTFT and OS as in the retrospective series from Essen. Most importantly, using TTFT as a read-out in a multivariate Cox regression model including Binet disease stage, CD38 and cytogenetic risk for the Essen cohort and IGHV status, lactate dehydrogenase (LDH), cytogenetic risk and high risk as defined by the study protocol in CLL1 series, progranulin had independent power predicting an unfavorable prognosis in both patient cohorts.

The molecular mechanisms underlying deregulated mRNA expression of *GRN* in CLL patients as compared to normal controls are not clear. We investigated the possibility that disrupted Pgrn expression may be caused by aberration of the *GRN* gene located at chromosome 17q21.31. Exploiting a panel of CLL cases which had been previously investigated for the presence of structural chromosomal abnormalities by high resolution SNP array profiling [31], we could not detect gains of chromosomal material at the *GRN* locus in any of the 55 individual CLL samples analyzed (data not shown). Interestingly, Jiao et al. [32] recently reported that miR-29b specifically binds to the 3′ untranslated region of *GRN* mRNA, leading to its down-regulation in miR-29b transfected HEK293 cells. MicroRNA-29b is heterogeneously expressed in CLL and has been demonstrated to be associated with an aggressive course of the disease [33]. Therefore, we employed qRT-PCR to measure miR-29b in CLL cells in relation to Pgrn protein levels in the same patients. There was a trend for an inverse correlation between miR-29b and Pgrn, however these results did not reach statistical significance (data not shown). Of note, time course analyses in a subgroup of eighteen CLL cases revealed that low risk patients show relatively stable Pgrn levels whereas high risk patients with progressive disease exhibited increasing Pgrn concentrations over time. However, a larger cohort of patients with sequential samples available needs to be analyzed in correlation to clinical and other prognostic factors to better understand the relationship between Pgrn levels and disease activity.

Progranulin contributes to many important biological processes and it is tempting to speculate about its potential role as a disease propagating factor in CLL. While to date no unique Pgrn-binding cell surface receptor has been identified [7], [8], recent work by Frampton et al. [23] showed that treatment of cholangiocarcinoma cells with recombinant Pgrn *in vitro* increased cell proliferation via an Akt-dependent mechanism. Along the same line Abrhale and coworkers [34] reported that Pgrn induces cell proliferation and confers aromatase inhibitor resistance in the breast cancer cell line MCF-7-CA. In contrast to these studies we could not detect a pro-survival effect of recombinant Pgrn in CLL suspension cultures which may be explained by differences in cell type and experimental conditions. Interestingly, Park et al. [35] recently showed that Pgrn may function as a critical soluble co-factor of TLR9 signaling in macrophages. As TLR9 signaling may contribute to the expansion of CLL cells *in vivo* through stimulation of the NF-κB pro-survival pathway we are currently investigating the possibility that Pgrn enhances the effects of TLR9 agonists on CLL cells.

In summary, these results along with our previously published gene expression data [2] indicate that Pgrn is a robust and reliable prognostic marker in CLL and therefore should be further tested in the context of prospective clinical trials. Finally, it will be interesting to investigate the molecular mechanisms underlying deregulated Pgrn expression and its functional implications for the biological behavior of CLL cells.

## Supporting Information

Figure S1 **Correlation of Pgrn plasma levels and** ***GRN*** **mRNA concentrations in individual CLL cases.** Samples from N = 16 patients were subjected to both *GRN* mRNA quantification using Affymetrix U133A microarrays and ELISA analysis of Pgrn plasma concentrations. mRNA expression values available from a previously published study (GSE4392) and Pgrn plasma levels were found to be correlated (R^2^ = 0.43, p = 0.0077, Spearman correlation). The regression line in the plot was produced by linear regression analysis.
(TIF)

Figure S2 **Secretion of Pgrn into cell culture media and survival of CLL cells in the presence of recombinant Pgrn.** Freshly isolated CLL cells from five individual patients were cultured as described in patients, materials and methods section. (A) ELISA revealed that CLL cells are capable of secreting Pgrn in a time-dependent fashion with large inter-individual differences. (B) Survival of CLL cells in the presence or absence of different concentrations of human recombinant Pgrn as determined by trypan blue exclusion. Data represent mean +/− SEM.
(TIF)

Figure S3 **Distribution of Pgrn levels in the study populations.** Pgrn concentrations were measured by ELISA in plasma and serum samples collected from CLL patients in Essen (A) and the CLL1 study cohort (B), respectively. The vertical lines represent the median Pgrn concentrations which were used as a cut-off to define patient subgroups with high *vs.* low Pgrn levels. For comparison of the clinical characteristics of the two cohorts refer to Tables 1 and 2. Note that the CLL patients from Essen exhibit statistically higher Pgrn levels than their counterparts from the CLL1 cohort (p<0.0001).
(TIF)

Figure S4 **Association of established prognostic markers and clinical outcome in the CLL cohort from Essen.** Kaplan-Meier curves depict the cumulative proportion of untreated patients with CLL (TTFT) grouped according to IGHV mutation status (A), cytogenetic risk (B), CD38 expression (C), ZAP-70 expression (D), Binet stage (E) and smudge cell percentage (F). Statistical analysis was conducted using the log-rank test.
(TIF)

Figure S5 **Comparison of Pgrn levels in heparin plasma as compared to serum collected simultaneously from the same patients.** The lines connect the symbols of five individual patients (A). Correlation of Pgrn plasma levels in plasma and serum samples collected simultaneously from the same patients (B). The regression line in the plot was produced by linear regression analysis.
(TIF)

Figure S6 **Association of established prognostic markers and clinical outcome in the CLL1 cohort.** Kaplan-Meier curves depict the cumulative proportion of untreated patients with CLL (TTFT) grouped according to IGHV mutation status (A), cytogenetic risk (B), thymidine kinase (C), study arm (D), LDH (E) and lymphocyte doubling time (F). Statistical analysis was conducted using the log-rank test.
(TIF)
