# Supplementary figures and images for: Progranulin Is a Novel Independent Predictor of Disease Progression and Overall Survival in Chronic Lymphocytic Leukemia
Source: PLoS One. 2013 Aug 23;8(8):e72107. doi: 10.1371/journal.pone.0072107 (PMC3751910; doi:10.1371/journal.pone.0072107)

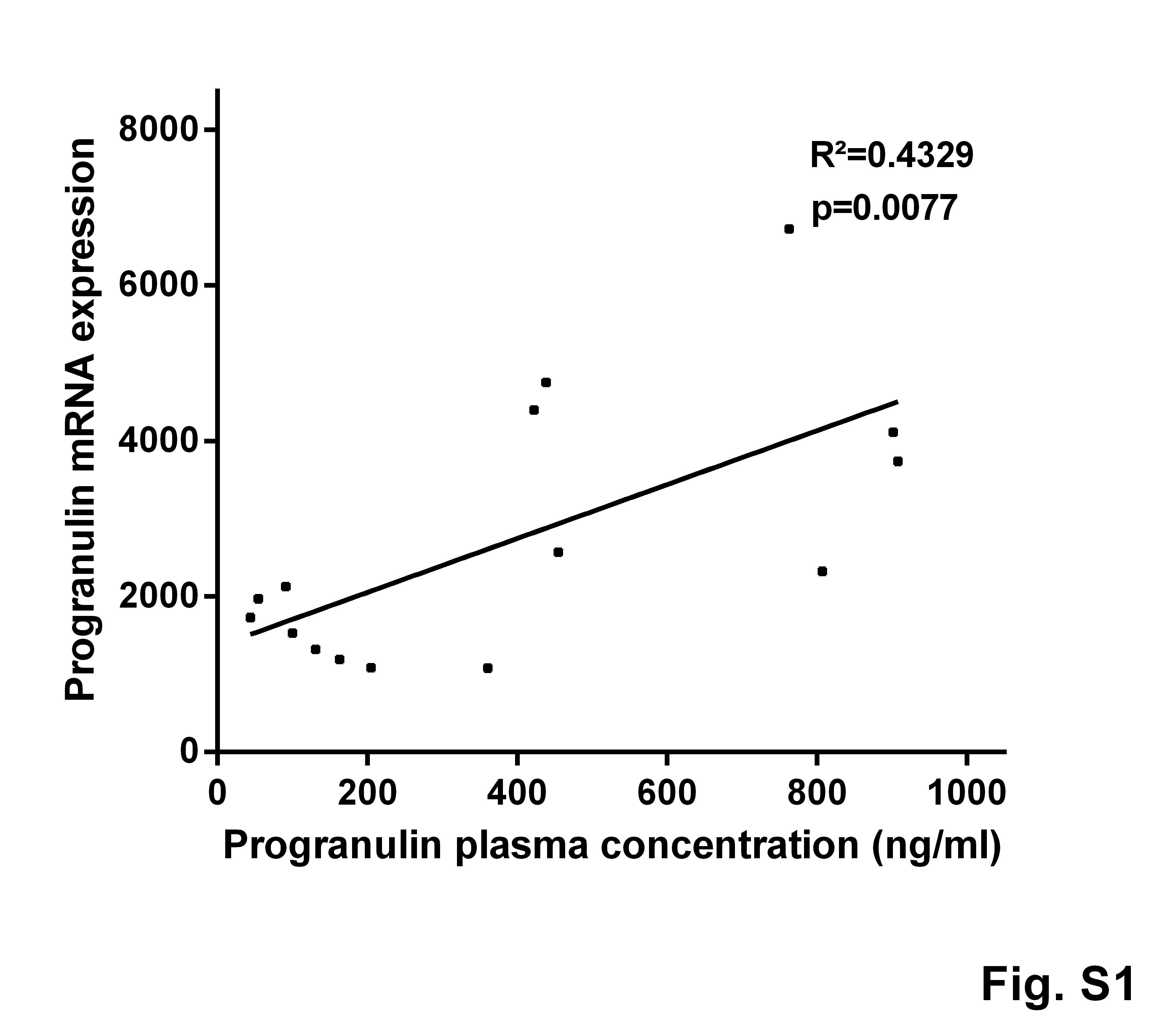

Supplement: Figure S1 — Correlation of Pgrn plasma levels and GRN mRNA concentrations in individual CLL cases. Samples from N = 16 patients were subjected to both GRN mRNA quantification using Affymetrix U133A microarrays and ELISA analysis of Pgrn plasma concentrations. mRNA expression values available from a previously published study (GSE4392) and Pgrn plasma levels were found to be correlated (R2 = 0.43, p = 0.0077, Spearman correlation). The regression line in the plot was produced by linear regression analysis. (TIF) [file pone.0072107.s001.tif]

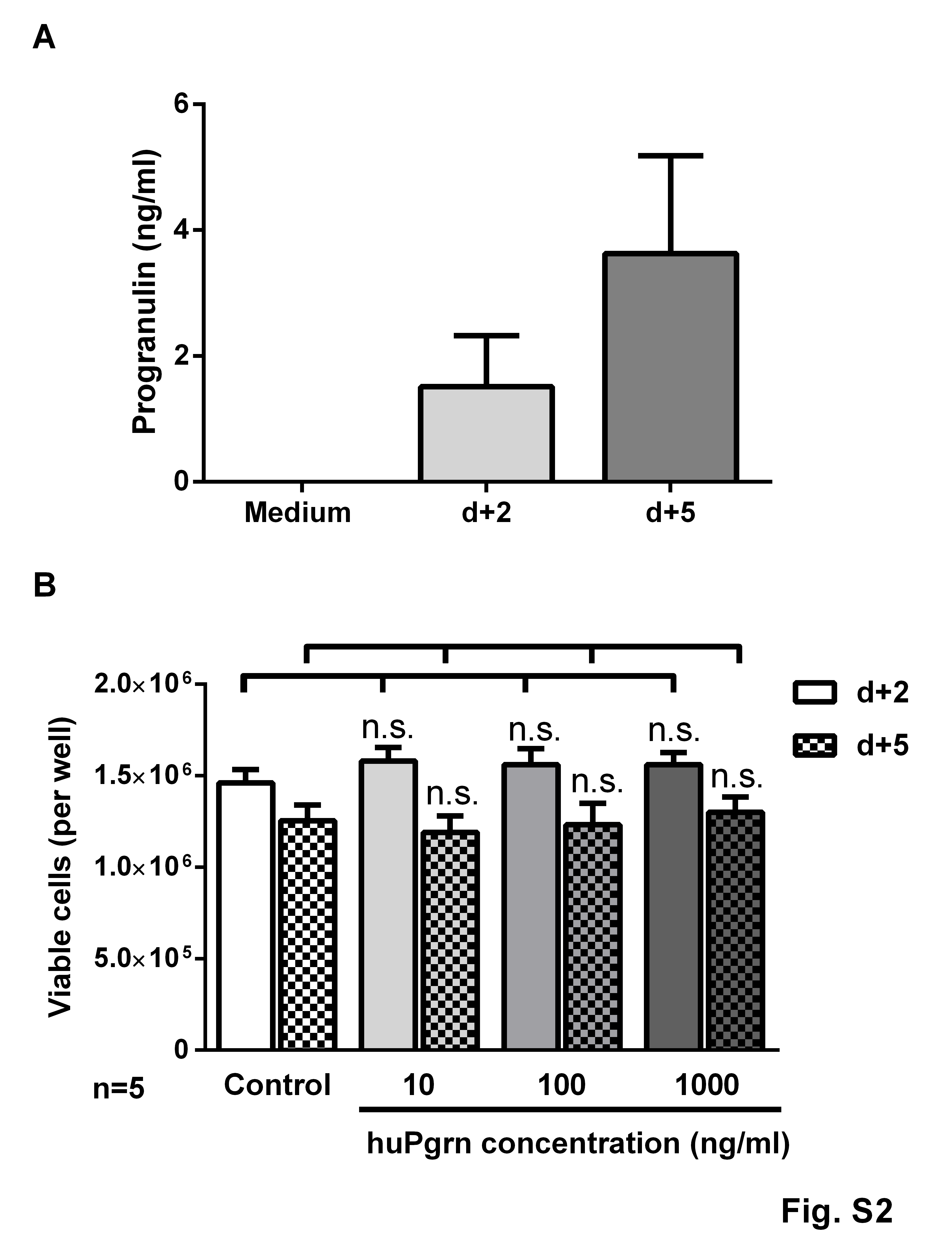

Supplement: Figure S2 — Secretion of Pgrn into cell culture media and survival of CLL cells in the presence of recombinant Pgrn. Freshly isolated CLL cells from five individual patients were cultured as described in patients, materials and methods section. (A) ELISA revealed that CLL cells are capable of secreting Pgrn in a time-dependent fashion with large inter-individual differences. (B) Survival of CLL cells in the presence or absence of different concentrations of human recombinant Pgrn as determined by trypan blue exclusion. Data represent mean +/− SEM. (TIF) [file pone.0072107.s002.tif]

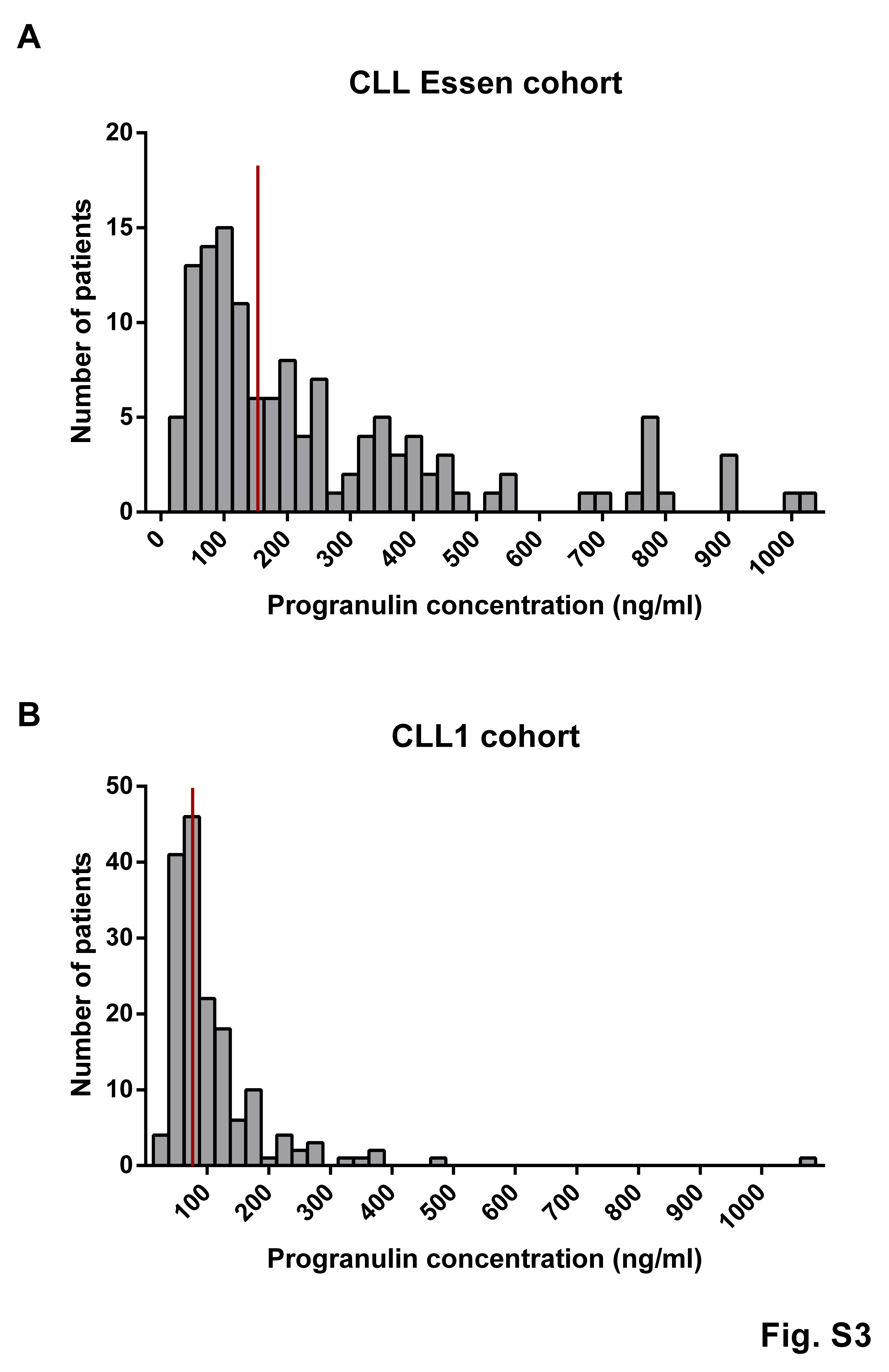

Supplement: Figure S3 — Distribution of Pgrn levels in the study populations. Pgrn concentrations were measured by ELISA in plasma and serum samples collected from CLL patients in Essen (A) and the CLL1 study cohort (B), respectively. The vertical lines represent the median Pgrn concentrations which were used as a cut-off to define patient subgroups with high vs. low Pgrn levels. For comparison of the clinical characteristics of the two cohorts refer to Tables 1 and 2. Note that the CLL patients from Essen exhibit statistically higher Pgrn levels than their counterparts from the CLL1 cohort (p<0.0001). (TIF) [file pone.0072107.s003.tif]

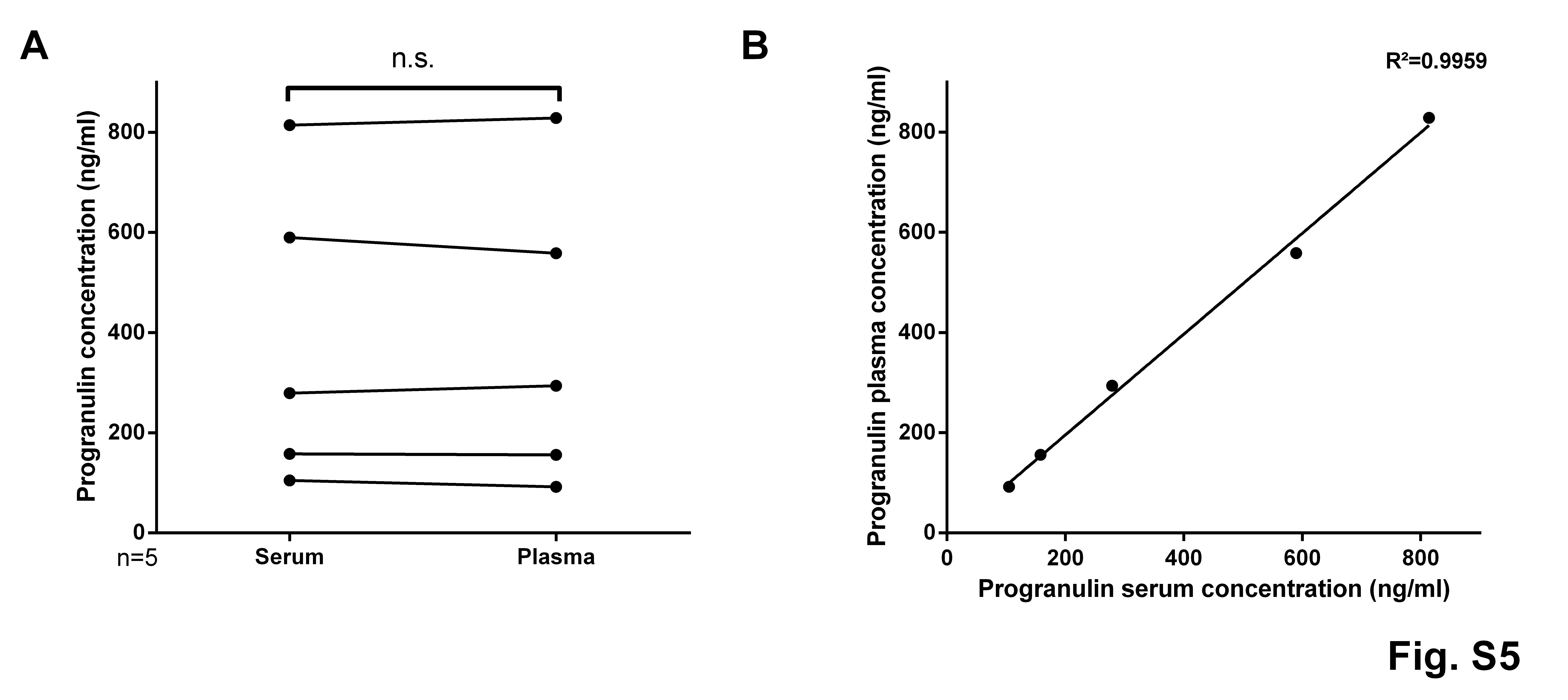

Supplement: Figure S5 — Comparison of Pgrn levels in heparin plasma as compared to serum collected simultaneously from the same patients. The lines connect the symbols of five individual patients (A). Correlation of Pgrn plasma levels in plasma and serum samples collected simultaneously from the same patients (B). The regression line in the plot was produced by linear regression analysis. (TIF) [file pone.0072107.s005.tif]
